# Landscape of chimeric RNAs in COVID-19 patient blood

**DOI:** 10.1016/j.gendis.2024.101348

**Published:** 2024-06-06

**Authors:** Samuel Haddox, Ping Wu, Sandeep Singh, Fujun Qin, Jack Engel, Andrea Kian, Syed Ahmad, Hui Li, Peng Wu

**Affiliations:** a Department of Biochemistry and Molecular Genetics, School of Medicine, University of Virginia, Charlottesville, VA 22908, USA; b Department of Gynecology and Obstetrics, Union Hospital, Tongji Medical College, Huazhong University of Science and Technology, Wuhan, Hubei 430030, China; c National Clinical Research Center for Gynecology and Obstetrics, Tongji Hospital, Tongji Medical College, Huazhong University of Science and Technology, Wuhan, Hubei 430030, China; d Cancer Biology Research Center (Key Laboratory of the Ministry of Education), Tongji Hospital, Tongji Medical College, Huazhong University of Science and Technology, Wuhan, Hubei 430030, China; e Computational Toxicology Facility, CSIR-Indian Institute of Toxicology Research, Lucknow, Uttar Pradesh 226001, India; f School of Basic Medical Sciences, Academy of Medical Sciences, Zhengzhou University, Zhengzhou, Henan 450001, China; g Department of Pathology, School of Medicine, University of Virginia, Charlottesville, VA 22908, USA

**Keywords:** Blood, Chimeric RNA, COVID-19, PTBP1, SARS-CoV-2

## Abstract

Despite the availability of efficacious vaccines, COVID-19 persists and our knowledge of how SARS-CoV-2 infection affects host transcriptomics remains incomplete. Transcriptome analysis, which has progressed our understanding of the patient response to SARS-CoV-2 infection, can be enhanced by considering chimeric transcript expression. Here we assess and characterize chimeric RNAs found in the whole blood of 178 COVID-19 patients. STAR-Fusion, SOAPfuse, and EricScript were used to detect chimeric RNAs resulting in over 30,000 predictions with approximately 500 high-confidence predictions that were found by more than one software and filtered based on exon annotations around the chimeric splice junction. GO term enrichment performed on the 5′ and 3′ parental genes of chimeric RNAs found in severe and critical patients resulted in pathways known to be affected in these patients, such as erythroid differentiation. Motif enrichment analysis of sequences proximal to chimeric splice junctions found in COVID-19 patients versus those found in GTEx whole blood revealed two RNA binding proteins previously implicated with coronavirus infection, PTBP1 and SFPQ. We discovered a chimeric RNA that correlated with COVID-19 disease status and appeared to be dependent upon a loss of PTBP1's function as a splicing repressor. Overall, we found over 350 novel COVID-19-specific chimeric RNAs not detectable in GTEx whole blood that may also serve as biomarkers for viral infection.

## Introduction

The coronavirus disease of 2019 (COVID-19) pandemic has had a detrimental impact on global health, yet the spread of severe acute respiratory syndrome coronavirus 2 (SARS-CoV-2) has been met with a momentous effort by collaborating researchers around the globe resulting in not only the successful development of multiple vaccines but also a vast accumulation of COVID-19 patient-derived next generation sequencing data. Analysis of COVID-19 patient-derived RNA sequencing (RNA-seq) was used to first discover the novel virus^1^ and has since provided invaluable insight into the genomic sequence of SARS-CoV-2 and its phylogenetic lineage.^2^^,^^3^ RNA-seq analysis of COVID-19 patient-derived peripheral blood has also revealed transcriptional signatures associated with excessive cytokine release and activation of p53-dependent apoptotic pathways, which may be causative for the lymphopenia experienced by COVID-19 patients.^4^ Transcriptome analysis has also been implemented into a multi-omic approach that has revealed neutrophil over-activation and metabolite levels that correlate with T cell dysfunction in critically ill COVID-19 patients.^5^ However, a more in-depth interrogation of the chimeric transcriptome of COVID-19-derived whole blood could further our understanding of COVID-19 pathology and the immune response to SARS-CoV-2, as well as reveal novel biomarkers for clinical usage.

Chimeric RNAs resulting from gene fusions have traditionally been associated with cancer. While gene fusions such as BCR-ABL1 in chronic myelogenous leukemia have served as effective therapeutic targets and cancer diagnostic markers,^6^ chimeric RNAs are not exclusively oncogenic and do not always result from transformative genomic rearrangements.^7^, ^8^, ^9^, ^10^, ^11^ Analysis of the Genotype-Tissue Expression (GTEx) dataset revealed over 2.5 million chimeric RNAs predicted to be found in various tissue types from approximately 550 individuals, highlighting the pervasiveness of chimeric RNA transcription in normal/healthy tissues.^8^^,^^12^^,^^13^ The propensity for chimeric RNA transcription in both diseased and healthy tissues along with the fact that detection of chimeric RNAs is typically excluded from standard gene expression pipelines motivates the characterization of chimeric RNAs to further our understanding of how the expanded functional transcriptome contributes to both pathology and normal physiology.

In this study, we used three different chimeric RNA prediction software to identify chimeric RNA transcripts in whole blood samples from COVID-19 patients. We filtered these predictions based on previously determined parameters known to be enriched in false positives before performing gene ontology and motif analysis on the resulting chimeric RNAs. By comparing the chimeric RNAs found in COVID-19 patients of different ages, sex, and disease status, along with previous chimeric RNA data from our GTEx analysis,^8^^,^^12^^,^^13^ we aim to discover chimeric RNA expression that correlates with SARS-CoV-2 infection and serve as potential biomarkers for patient prognosis.

## Materials and methods

### Ethics statement

This study was reviewed and approved by the Institutional Review Board of Tongji Hospital, Tongji Medical College, Huazhong University of Science and Technology, China (TJ-IRB20200405). Signed informed consent was provided by all enrolled patients and blood remaining from standard diagnostic tests was used for sequencing to prevent the need for additional collections from patients.

### Data acquisition

We have generated PE-100bp RNA-seq data for COVID-19 patient whole blood and deposited it onto the European Nucleotide Archive (ENA) at EMBL-EBI under project accession number PRJEB43380 (https://www.ebi.ac.uk/ena/browser/view/PRJEB43380).^5^ GTEx chimeric RNA data was produced by Singh et al from RNA-seq data provided by the GTEx project (V6 dbGaP Accession phs000424.v6.p1).^8^^,^^12^^,^^13^ Additionally, RNA-seq fastq files from Liu et al (2018; GEO Accession: GSM2842780) were used.^29^

### Bioinformatic prediction of chimeric RNAs

GRCh38 was used as the reference genome and paired-end RNA-seq fastq files were analyzed with three different chimeric RNA prediction software. Ericscript was used with default parameters with additional filtering of false positives as described in the methods of Singh et al.^14^ Soapfuse was used with default parameters.^15^ Star-Fusion was also used for chimeric RNA prediction.^16^ Here we adjusted Star-Fusion for increased sensitivity and increased detection of chimeras that resulted from transcriptional read-through. Adjusted parameters included STAR_max_mate_dist 1000, STAR_chimMultimapNmax 50, max_promiscuity 50, min_pct_dom_promiscuity 10, aggregate_novel_junction_dist 5, min_novel_junction_support 1, min_spanning_frags_only 0, min_alt_pct_junction 5, and min_FFPM 0.01. Chimeric predictions were filtered to keep only those that were found using more than one software.

Additionally, chimeric RNA predictions were classified by localizing chimeric junction coordinates to exon boundaries as described in Babiceanu et al.^17^ Chimeric RNA predictions classified as M:M (Middle to Middle; both the chimeric splice donor and acceptor coordinates map to the middle of annotated exons) were filtered out. For chimeric RNAs to be classified as COVID-19 specific, they could not be found in more than four GTEx blood samples. Alternatively, if predicted chimeric RNAs were found to be significantly different between patient age, sex, or disease status groups using the chi-square test, they were included in later analyses.

### Statistical analysis

Because the detection capacity of each chimera varied between prediction software, we used Agrep^18^ to search for chimeric junction sequences directly in each sample fastq file. Agrep determined frequencies were used with chi-square analysis to look for chimeric RNA associations with disease status, age, or sex.

### Chimeric RNA profiling

Agrep was used to search for reads that provided evidence for the presence of each recurrent chimeric RNA in each sample. A binary matrix was created with a row for each sample and a column for each recurrent chimera, and each cell was given a value of either 1 or 0 representing the presence or absence of the chimera in the respective sample. We also created a binary matrix for the parental genes using STAR alignments with cufflinks to determine the FPKM of each parental gene. We determined that the lowest expression FPKM value for the Agrep detected chimera was 0.074 and used this as the cutoff for each parental gene. If a parental gene's FPKM ≥0.074, it was given a value of 1, otherwise, it was given a value of 0. This ensures we are determining the parental genes' binary value the same as the chimeras'. A separate binary matrix was created for 5′ parental genes and 3′ parental genes.

### Motif and gene ontology analysis

Using the Gapped Local Alignment of Motifs (GLAM2) tool from the MEME SUITE with default parameters, we assessed 5′ and 3′ parental genes 200 bps upstream and downstream of the chimeric junctions to find enriched motifs.^19^^,^^20^ Next, the Tomtom tool from MEME SUITE was used with default parameters and a database of known RNA binding protein motifs to determine the associated RNA binding proteins (RBP).^20^, ^21^, ^22^

Using the Gorilla tool, gene ontology (GO) terms were predicted for the 5′ and 3′ parental genes of chimeras with all annotated genes in GRChh38 as background.^23^ Only the top 10 most significant hits were reported.

### PCR and sanger sequencing

The total RNA was extracted using the miRNeasy Mini Kit (217004, QIAGEN, Germany). cDNA was synthesized using the RT-PCR Kit (HF001-01, Vazyme, Nanjing) according to the manufacturer's instructions. Bio-Rad CFX96 Real-Time System (Bio-Rad, USA) was used to perform PCR with HiScript ⅡQ RT SuperMix (R223-01, Vazyme, Nanjing). The primers were synthesized by Tsingke Biotechnology Co., Ltd (Wuhan, China), and the sequences of the primers are displayed in Table S1. The amplified PCR products were separated by DNA electrophoresis and submitted for Sanger sequencing to validate the junction sequence of chimeric RNA candidates.

## Results

### COVID-19 patient whole blood chimeric transcriptome

**Figure 1:**
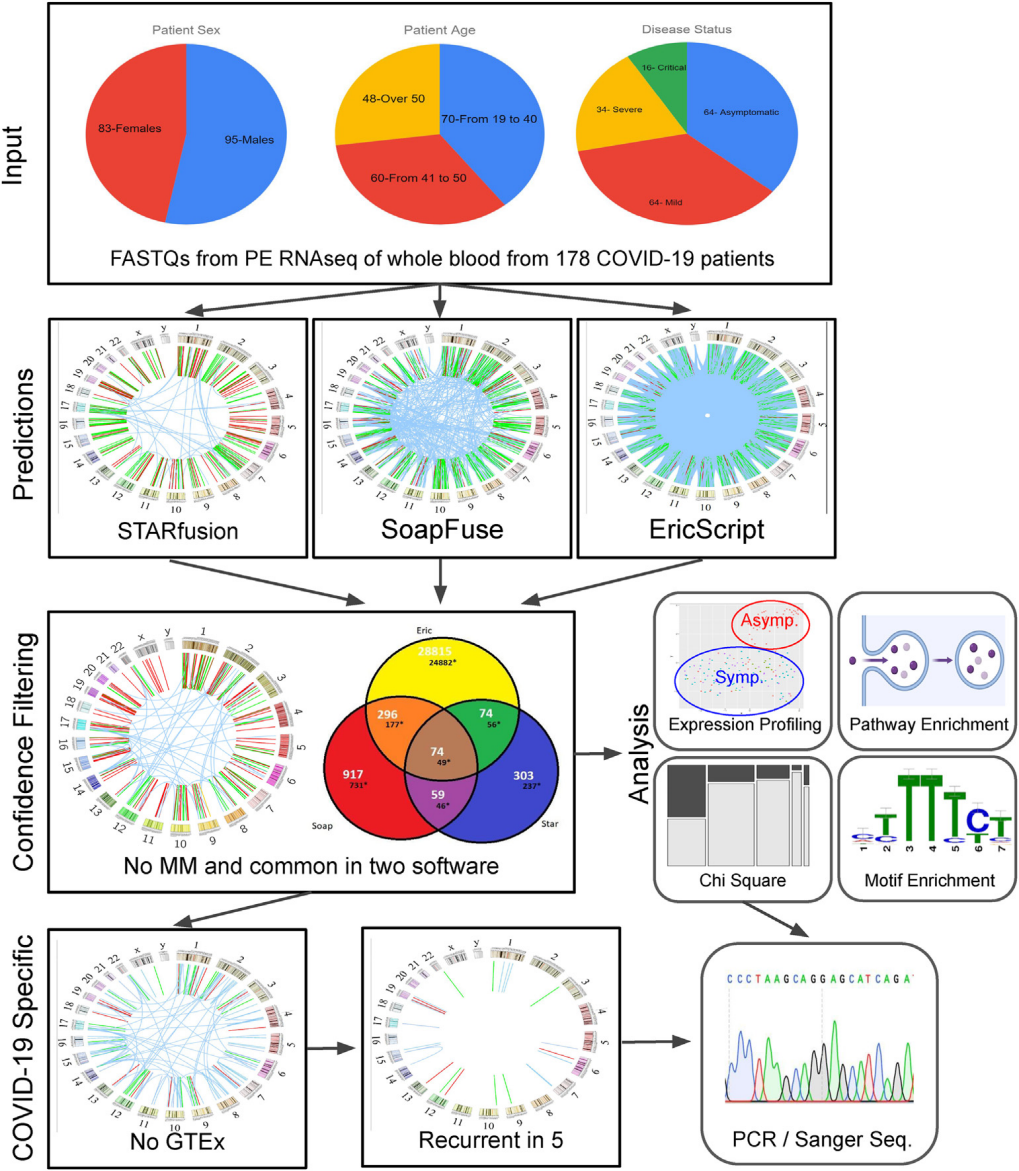
Overall workflow of the study. COVID-19 patient whole blood sequencing data was analyzed for chimeric RNA using STARfusion, SoapFuse, and EricScript. Predictions found by at least two software and those containing no middle-to-middle (M:M) chimeric splice junctions were kept. All high-confidence predictions were used for downstream analysis. Downstream analysis included expression profiling, pathway enrichment, motif enrichment, and chi-square contingency tables. High confidence predictions were also further filtered to remove chimeras found in GTEx samples and spurious results found in less than five patients. Finally, PCR and Sanger sequencing were used to validate recurrent, COVID-19-specific chimeras, and the chimeras found to have an association with age, sex, or disease status.

As shown in the flowchart of the analysis pipeline (Fig. 1), we assessed a cohort of 178 COVID-19 patients consisting of 83 females and 95 males ranging from 19 to 88 years of age who were categorized by disease status (asymptomatic, mild, severe, or critical). We utilized STAR-Fusion, SOAPfuse, and EricScript to predict the presence of chimeric RNAs in the transcriptome of whole blood. It has previously been demonstrated that EricScript may generate false positives when chimeric junction sequences are similar to non-chimeric transcript references and therefore EricScript predictions are only considered after BLAT-filtering.^8^ The totals of chimeric predictions from STAR-Fusion, SOAPfuse, and BLAT-filtered-EricScript were 532, 4143, and 70,529 respectively, and are shown in Tables S1–3. As expected, Ericscript generated the most predictions; however, these tend to contain frequent false positives. Contrarily, STAR-Fusion has more stringent filtering and tends to generate far less chimeric RNA predictions. In the past, we have found that predictions with M:M chimeric splice junctions are enriched in false positives; therefore, we only considered chimeric RNA predictions in which the junction sequences aligned to either the 5′ or 3′ end of an annotated exon.^17^ The M:M filtered chimeric RNA totals from STAR-Fusion, SOAPfuse, and BLAT-filtered-EricScript were 489, 1382, and 29,204 respectively, and are shown in Tables S4–6. To strengthen the confidence in our chimeric RNA predictions, only chimeras predicted by two or more of the implemented software were considered. This resulted in 503 chimeric RNA predictions representing 328 unique parental gene pairs as shown in the Venn diagram in Figure 1.

### Characterization and distribution of chimeric RNAs

**Figure 2:**
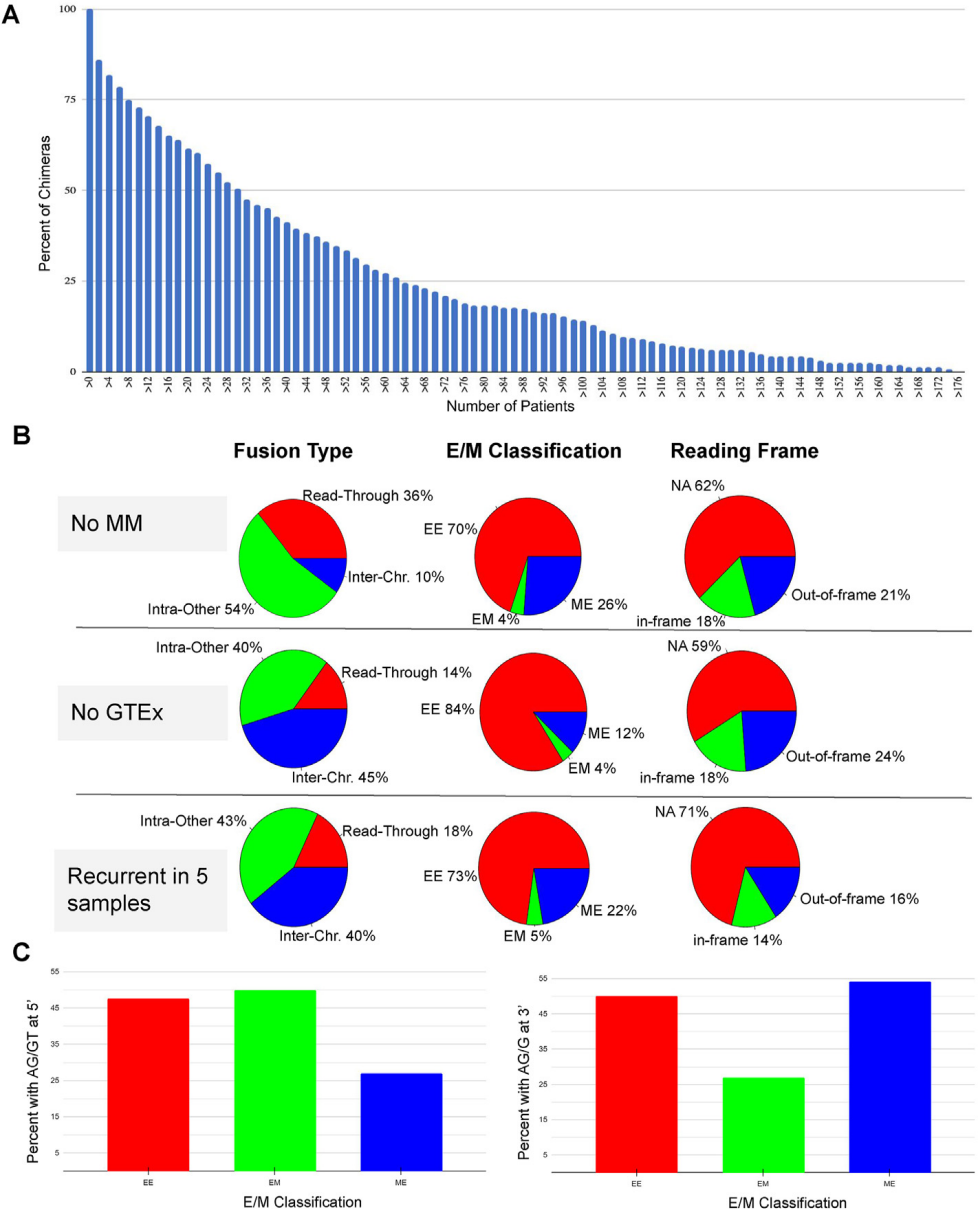
Frequency and categories of chimeric RNAs. **(A)** The percentage of chimeric RNAs plotted against their frequencies. **(B)** The distribution of chimeric RNAs after removal of middle-to-middle (M; M) chimeras, removal of GTEx chimeras, and removal of chimeras found in less than five samples based on different categories. **(C)** Percentage of chimeric RNAs harboring the canonical splicing donor sequence (AG/GT) at the 5′ junction (left) and percentage of chimeric RNAs harboring canonical splicing acceptor sequence (AG/G) at the 3′ junction (right).

The distribution of chimeric RNA frequencies is shown in Figure 2A. We characterized chimeric RNAs by exon-based alignment of junction sequences (E/M classification), relative parental gene location (Fusion Type), and putative fusion protein-coding potential (Reading Frame) at each level of filtering as previously described.^8^ The characterization and distribution of chimeric RNAs are shown in Figure 2B. Starting with M:M filtered chimeric RNAs that are recurrent in two or more software, there are 503 unique chimeric RNAs (Table S7); 90% were generated from intra-chromosomal transcriptional processes (including intra-other and read-through chimeras) and 70% had junction sequences aligning with exon boundaries from both parental genes (E:E). After filtering out the chimeric RNAs previously detected in GTEx samples (Table S8), there remained 359 unique chimeric RNAs; 45% of the chimeric RNAs were generated from inter-chromosomal processes and 16% had junction sequences aligning only to a single parent gene's annotated exon boundaries (E:M or M:E). The out-of-frame portion appeared to be reduced by twice as much as the in-frame portion when applying the recurrent in 5 samples filter (Table S9). The Fusion Type category exhibited the most notable change in distribution when the GTEx filter was applied, with a dramatic reduction in the intra-chromosomal portion of chimeric RNA predictions. Chimeric RNA type, alignment to annotated exon boundaries, and frame-shift status did not significantly change between disease status (Fig. S1).

### Assessment of canonical splicing at chimeric junctions

Next, we evaluated the presence of canonical splicing in chimeric RNA predictions. We independently assessed both the 5′ parental gene and 3′ parental gene splicing and compared this with exon-based alignment classification of the junction sequence as shown in Figure 2C. For 5′ gene chimeric junctions, we searched for AG/GT nucleotides flanking the splice donor site. For the 3′ gene chimeric junction we searched for AG/G nucleotides flanking the splice acceptor site. As expected, the E:E class of chimeras showed more robust canonical splicing at both the 5′ and 3′ junction sites, the E:M class exhibited more canonical splicing at the 5′ junction than the M:E class, and the M:E class demonstrated more canonical splicing at the 3′ junction than the E/M class. Chimeric splice junctions that involved annotated exon boundaries had a higher frequency of canonical splicing than chimeric splice junctions involving unannotated exon boundaries. It is, however, unexpected that the critical disease group has notably diminished proportions of 5′ canonical splicing and 3′ canonical splicing in the M:E and E:M chimeras respectively while maintaining relatively equal proportions of canonical splicing motifs in E:E chimeras at both the 5′ and 3′ splice sites (Fig. S2).

### Chimeric RNA profiling

**Figure 3:**
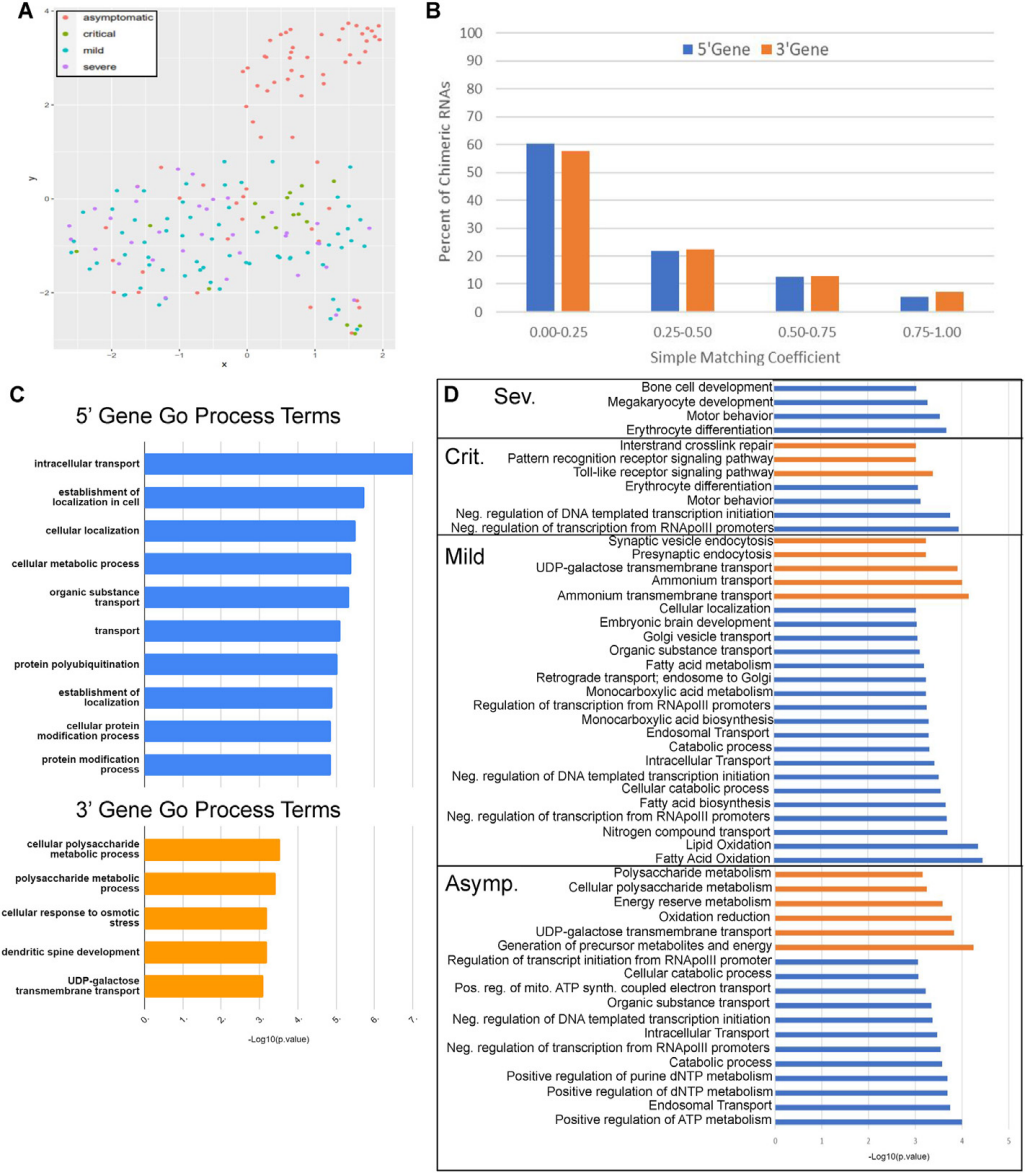
Chimeric RNA expression and parental gene characterization. **(A)** Uniform Manifold Approximation and Projection (UMAP) clustered asymptomatic cases away from symptomatic COVID-19 cases. **(B)** Simple matching coefficients (SMC) demonstrated that chimeric RNAs largely had different binary expression profiles than parental genes. **(C)** Gene ontology (GO) molecular process terms for parental genes of all chimeras. **(D)** GO molecular process terms for parental genes of chimeras from each disease group. 3′ gene terms and 5′ gene terms are indicated in orange and blue respectively.

Chimeric transcripts have been used as diagnostic biomarkers, and recently have been shown to be tightly associated with specific cell and tissue types.^8^^,^^30^ We therefore sought to answer whether chimeric RNA transcripts found in whole blood samples taken from COVID-19 patients were unique to SARS-CoV-2 infection or to a particular COVID-19 patient status, sex, or age group. Chimeric RNA profiles were created by designating their presence or absence (binary profile) in each patient sample and using Uniform Manifold Approximation and Projection (UMAP) as a dimension reduction technique to visualize unbiased clustering of samples. UMAP was unable to cluster samples by age or sex; however, as seen in Figure 3A, most of the asymptomatic samples clustered together away from mild, severe, and critical samples. There appears to be an overlap of all symptomatic groups with critical samples clustered more tightly together. While the UMAP analysis was not able to distinguish binary chimeric RNA expression profiles between each disease status, it does appear that some chimeric RNA expression may be associated with the symptomatic response to SARS-CoV-2 infection. We then investigated if chimeric RNA expression reflects parental gene expression. We created a binary matrix of parental gene expression to compare with the binary chimeric transcript matrix by determining a simple matching coefficient (SMC). SMC values range from 0 to 1, as SMC values increase the similarity between chimeric transcript expression and their respective parental transcript expression also increases. As shown in Figure 3B, approximately 60% of the chimeric RNAs had significantly different binary expression profiles than their respective parental genes. Less than 10% of the chimeric RNAs had a very similar (SMC ≥0.75) binary expression profile to their respective parental genes. Taken together, this indicates that the expression of chimeric RNAs in whole blood differs between symptomatic and asymptomatic COVID-19 patients and that the binary expression profiles of chimeric and parental transcripts are not similar, supporting that chimeric RNAs represent the result of another layer of transcriptional control.

### Gene ontology enrichment analysis

As in our previous GTEx analysis, recurrent non-M:M predictions were used for gene set enrichment analysis with Gorilla to identify GO terms associated with 5′ and 3′ parental genes.^8^ Results are shown in Figure 3C. In general, 5′ parental genes demonstrated more significant enrichments in GO terms for cellular processes than 3′ parental genes. One recent report suggests that when assessing the parental genes of read-through chimera, 5′ parental genes tend to be more enriched in processes associated with the observed phenotype than 3′ parental genes. Our results may reflect this as our chimeric predictions are largely read-through (neighboring genes) or intra-chromosomal, which may also result from transcriptional read-through of non-neighboring genes nearby. This may be due to transcriptional machinery targeting the 5′ gene regulatory regions while co-transcriptional dysregulation leads to aberrant transcriptional read-through into “non-targeted” genes.^40^ GO term processes associated with 5′ parental genes include intracellular transport and establishment of localization in the cell. GO term processes associated with 3′ genes include polysaccharide metabolism and cellular response to osmotic stress.

We then separated samples into groups based on their disease status (asymptomatic, mild, severe, and critical) and performed GO term enrichment for cellular processes on each, using 5′ and 3′ parental genes from chimeric RNAs found in more than one sample of that group (Fig. 3D). All groups but critical demonstrated 5′ gene enrichment in processes associated with the negative regulation of DNA-templated transcription. The critical and severe groups both showed 5′ gene enrichment in motor behavior and erythrocyte differentiation processes while the asymptomatic and mild groups did not. The asymptomatic group showed the most significant 5′ gene enrichment in ATP and nucleotide metabolic processes. The mild group demonstrated a unique 5′ gene enrichment in lipid oxidation. The critical group was the only group to show 5′ gene enrichment in bone and megakaryocyte development processes. As for the 3′ gene enrichment, there was no overlap in processes between disease status groups and the critical group did not show any GO term enrichment. The most significantly enriched asymptomatic 3′ gene process is associated with the generation of precursor metabolites and energy. Ammonium transmembrane transport processes were most enriched in 3′ gene processes for the mild group. The severe group has 3′ gene enrichment in toll-like receptor signaling and interstrand crosslink repair pathways.

Interestingly, our GO term analysis appears to corroborate with another recent study of the chimeric transcriptome of severe COVID-19 patient whole blood that found an enrichment of genes contributing to chimeric transcription in pathways associated with synaptic vesicles, metabolic, cellular localization, cellular response to oxidative stress, and immune system response processes.^32^

### Chimeric junction sequence enrichment and RNA binding protein motif analysis

**Figure 4:**
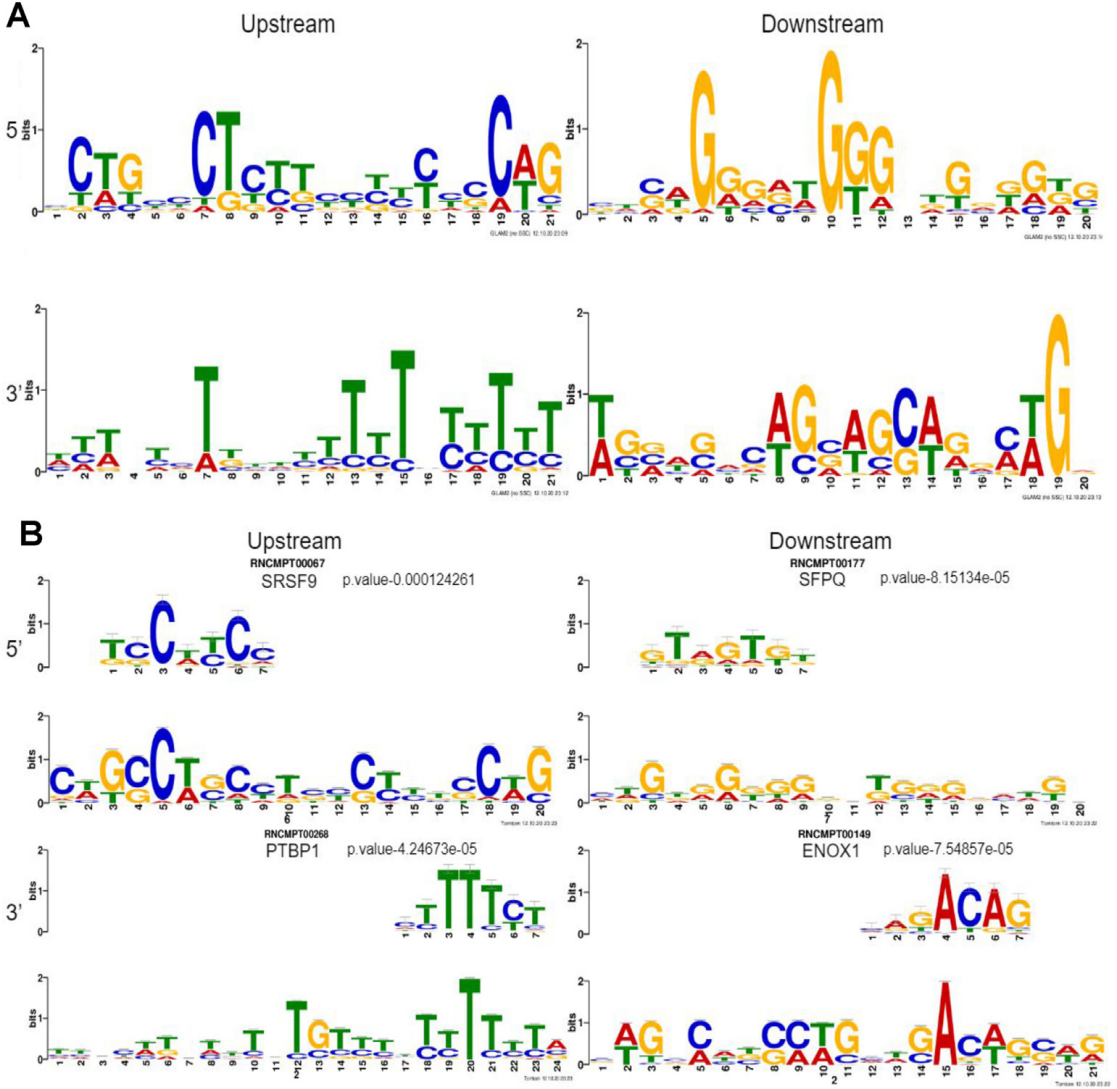
RNA binding protein motif enrichment analysis with GLAM and TomTom. **(A)** Top enriched DNA sequences upstream and downstream of the 5′ and 3′ breakpoints. **(B)** The sequence logos above show the most enriched RNA binding protein motifs (SRSF9, SFPQ, PTBP1, and ENOX1) found above their top enriched reference sequences. SRSF9, serine and arginine rich splicing factor 9; SFPQ, splicing factor proline and glutamine rich; PTBP1, polypyrimidine tract binding protein 1; ENOX1, ecto-NOX disulfide-thiol exchanger 1.

To assess the enrichment of sequence motifs flanking chimeric junction sites, we used upstream and downstream genomic sequences of 200 bp length from both the 5′ and 3′ chimeric junction sites (non-M:M chimeras) as input for GLAM2. The highest scoring upstream and downstream sequence enrichments for the 5′ and 3′ chimeric junction sites are presented in Figure 4A. The output from GLAM2 was then used as input for Tomtom to identify potential RBP motifs associated with the enriched sequences. Several RBPs were identified for enriched motifs in the upstream and downstream regions flanking both the 5′ and 3′ junction sites. The RBPs with the highest *P*-values for each region are shown in Figure 4B (other significant RBP binding motif enrichments are shown in Figs. S3A–D). Serine and arginine rich splicing factor 9 (SRSF9), with a *P*-value of 1.2 × 10^−4^ was identified to have an RNA binding site motif found in sequences enriched in the upstream region of 5′ parental gene chimeric junction sites; splicing factor proline and glutamine rich (SFPQ), with a *P*-value of 8.2 × 10^−5^ was identified to have an RNA binding site motif found in sequences enriched in the downstream region. Polypyrimidine tract binding protein 1 (PTBP1), with a *P*-value of 4.2 × 10^−5^ was identified to have an RNA binding site motif found in sequences enriched in the upstream region of 3′ parental gene chimeric junction sites; ecto-NOX disulfide-thiol exchanger 1 (ENOX1), with a *P*-value of 7.5 × 10^−5^ was identified to have an RNA binding site motif found in sequences enriched in the downstream region. SRSF9 was also identified as having the top enriched RNA binding protein motif upstream of the 5′ breakpoint in our previous GTEx whole blood analysis; while RNA binding motif protein 5 (RBM5) had the top enriched RNA binding protein motif downstream of the 5′ breakpoint.^8^ For the 3′ breakpoint of GTEx whole blood chimeras, ENOX1 and poly (rC)-binding protein 2 (PCBP2) were found to have the top enriched RNA binding protein motif upstream and downstream, respectively.^8^ The RNA binding protein motifs of SFPQ and PTBP1 were found to be the top enriched RBP motifs in COVID-19 patient chimeras and not in GTEx whole blood chimeras. SFPQ, previously found to be involved in influenza transcription, was more recently found to interact with the SARS-CoV-2 genome and contribute to viral RNA amplification.^24^^,^^25^ PTBP1 has previously been implicated in other coronavirus infections, where both PTBP1 RNAi knockdown and PTBP1 translocation from the nucleus to viral RNAs in the cytoplasm correlated with increased coronavirus production.^26^ PTBP1 was also found to interact specifically with SARS-CoV-2 and the production of SARS-CoV-2 proteins in cultured lung epithelial cells was shown to increase the production of PTBP1 as well as mRNA transcripts dependent on PTBP1 splicing during pre-mRNA processing.^25^^,^^27^^,^^28^

### PCR/sanger sequencing validation of select chimeric RNAs

**Figure 5:**
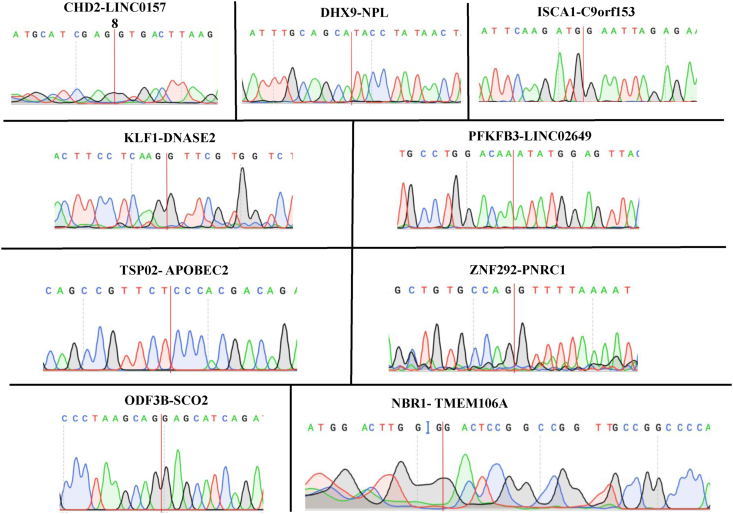
Experimental validation of selected chimeric RNAs. PCR primers flanking the chimeric junction were used to amplify the fragment across the fusion junction. Sanger sequencing was used to confirm the junction sequence.

Chimeras showing a significant correlation with disease status, age, or sex were selected based on chi-square analysis; alternatively, non-M:M, recurrent COVID-19 chimeras were still considered for follow-up validation if not found to be recurrent in the normal/healthy blood from previous GTEx reports.^8^ 10 out of 18 chimeric RNAs were successfully amplified via reverse transcriptase with random hexamers and subsequent PCR. A summary of the chimeric RNA chi-square analysis and validation results is shown in Table 1. Sanger sequencing traces showing the junction sequences of select validated chimeric RNAs are shown in Figure 5.

**Table 1:** Chimeric RNA PCR validation and clinical correlation.

Chimeric RNA	Pval_Sex	Pval_Age	Pval_Status	Validated
ARRDC2-R3HDM4	3.64E-01	**4.07E-02**	4.92E-01	No
CHD2-LINC01578	1.00E-00	**1.87E-02**	**3.96E-04**	**Yes**
COQ8B-NUMBL	7.65E-01	**8.42E-03**	**3.95E-02**	No
DENND4A-MARK3	3.07E-01	**3.89E-02**	6.67E-01	No
DHX9-NPL	3.69E-01	7.15E-02	**1.84E-02**	**Yes**
ISCA1-C9orf153	4.95E-01	1.42E-01	**4.10E-02**	**Yes**
KDM7A-MKRN1	9.14E-01	3.10E-01	4.42E-01	No
KLF1-DNASE2	8.38E-01	8.66E-01	**1.79E-07**	**Yes**
KRT128P-KRT73	2.41E-01	8.02E-01	**1.89E-04**	No
LAMTOR5-AS1-RBM15	7.05E-01	9.12E-01	8.50E-02	No
LMAN2-MXD3	6.33E-01	9.43E-01	**5.35E-03**	**Yes**
NBR1-TMEM106A	3.42E-01	6.62E-01	**8.34E-07**	**Yes**
ODF3B–SCO2	1.75E-01	5.20E-01	**1.68E-12**	**Yes**
PFKFB3-LINC02649	6.47E-01	7.28E-01	**1.58E-12**	**Yes**
RHD-TMEM50A	8.95E-01	7.61E-01	**7.15E-04**	No
TMEM272-DHRS12	3.67E-01	4.38E-01	**7.91E-13**	No
TSPO2-APOBEC2	6.59E-01	5.99E-01	**1.01E-02**	**Yes**
ZNF292-PNRC1	8.41E-01	4.14E-01	**5.94E-03**	**Yes**

Chimeric RNA PCR validation and Chi Square results for COVID-19 patient sex (Pval_Sex), age (Pval_Age), and disease status (Pval_Status). Significant P values are in bold and underlined text.

### Validation of PTBP1's contribution to COVID-19 patient blood chimeric RNAs

**Figure 6:**
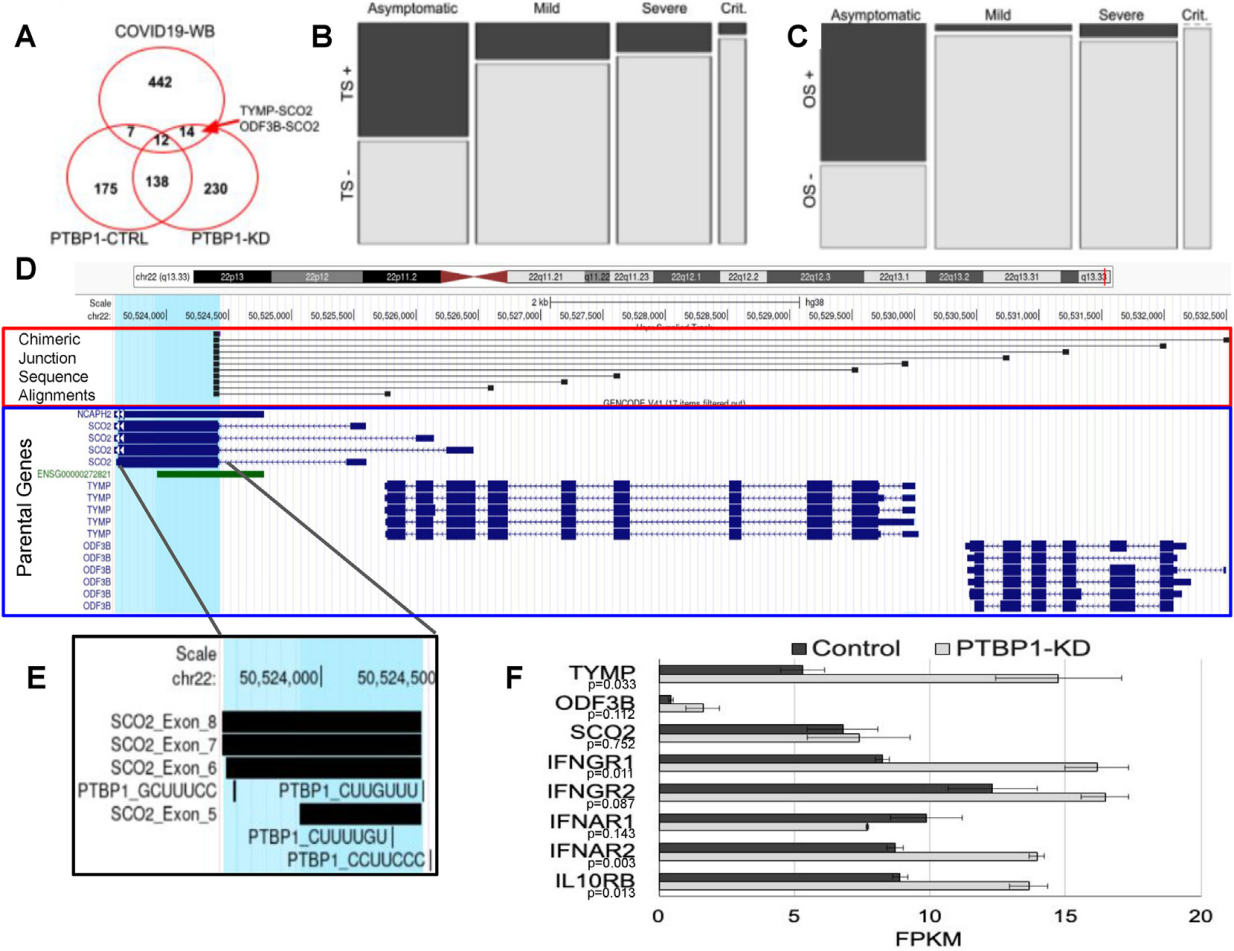
PTBP1 associated chimeric RNAs. **(A)** OS and TS isoforms were among 14 chimeras found only in COVID-19 whole blood and PTBP1 knockdown. **(B)** Mosaic plot for TS detection in COVID-19 patients by disease status. **(C)** Mosaic plot for OS detection by disease status. **(D)** Alignment of chimeric junction sequences to their parental genes for all detected PTBP1-dependent chimeras around the TS/OS locus. **(E)** PTBP1 RNA binding motifs proximal to the terminal SCO2 exon. **(F)** Gene expression of interferon (IFN) signaling and chimeric parental genes in CD34 primary cells treated with erythropoietin (EPO) (control *vs*. PTBP1 knockdown). PTBP1, polypyrimidine tract binding protein 1; SCO2, synthesis of cytochrome c oxidase 2; OS, ODF3B (ciliary microtubule associated protein 1B)–SCO2; TS, TYMP (thymidine phosphorylase)-SCO2.

Based on literature corroborating the interaction between coronaviruses and polypyrimidine tract binding proteins, namely PTBP1,^25^, ^26^, ^27^, ^28^ we aimed to validate whether modulation of PTBP1 expression in human blood cells could influence the generation of chimeric RNA transcripts, particularly those found from our analysis of COVID-19 patient whole blood. Accordingly, we found the raw RNA sequencing data from Liu et al (GEO Accession: GSM2842780) consisting of primary CD34 derived undergoing erythropoiesis and treated with either a mock shRNA lentivirus or one targeting PTBP1.^29^ Using Star-Fusion with the above-mentioned parameters, we predicted chimeric RNAs in all four samples. We then compared chimeric RNAs found from the PTBP1 knockdown and control groups with those found in the COVID-19 patients' whole blood using STAR-Fusion with the same parameters. The ODF3B (ciliary microtubule associated protein 1B)–SCO2 (synthesis of cytochrome c oxidase 2) (OS) and TYMP (thymidine phosphorylase)-SCO2 (TS) chimeric isoforms made up the majority of the 14 chimeras found only in the PTBP1 knockdown and COVID-19 samples (Fig. 6A). The OS and TS transcript exhibited a strong correlation with disease status using chi-square contingency table (Fig. 6B, C). Figure 6D depicts OS and TS chimeric splice junctions detected in PTBP1 knockdown and COVID-19 samples. PTBP1 RNA binding motifs were present in TS and OS transcripts before, on, and straddling the terminal SCO2 exon containing the chimeric splice acceptor site (Fig. 6E). This indicates a potential inhibitory role of PTBP1 on the inclusion of the terminal SCO2 exon.^31^ It is interesting that PTBP1 knockdown in primary CD34-derived erythroid cells not only generated OS and TS alternatively spliced chimeric transcripts, but it also led to ∼3-fold increase in expression of TYMP and ODF3B total transcripts (Fig. 6E). TS and OS transcript expression were more frequently found in asymptomatic COVID-19 patients than any other group. Based on the transcriptomic analysis of the COVID-19 patient cohort by Wu et al, interferon (IFN) signaling is significantly increased in asymptomatic patients.^5^ The only abundantly expressed IFN and IFN receptor genes abundantly expressed in the CD34-derived erythropoietic cell culture used in the PTBP1 experiment were IL10RB, IFNAR1, IFNAR2, IFNGR1, and IFNGR2.^5^^,^^29^ Most of the abundantly expressed IFN-associated genes demonstrated elevated expression under PTBP1 knockdown treatment (Fig. 6E) like that of the asymptomatic group of COVID-19 patients. PTBP1 appears to not only play a role in generating chimeric RNAs like TS but it also appears to be involved in IFN signaling, which correlates with the COVID-19 asymptomatic patient data.

## Discussion

It is known that chimeric RNAs arising from rearrangements at the genomic level, cis-splicing between adjacent genes (cis-SAGe), and RNA trans-splicing may contribute to neoplastic phenotypes^6^^,^^9^^,^^30^^,^^43^; however, except for rearrangements in immunoglobulin antigen-receptor associated genes, we have only more recently begun to appreciate the involvement of chimeric RNAs in a non-oncogenic context. Based on previous works in establishing a baseline of chimeric RNA transcription in GTEx, we were able to compare “normal” chimeric RNAs found in the whole blood of GTEx donors with those found in the whole blood of patients infected with SARS-CoV-2 and characterize SARS-CoV-2 specific chimeras. We found 359 chimeric RNAs in our SARS-CoV-2 analysis that were not present in the GTEx predictions. When applying the GTEx filter, we noticed that the bulk majority of filtered-out GTEx chimeras were intra-chromosomal/read-through. Furthermore, we noticed that chimeras removed by the GTEx filter were mostly M:E, which is surprising due to GTEX chimeras being predominantly classified as E:M.^8^ Of the 359 COVID-19 patient-specific chimeras, 47 were found to be recurrent in five or more samples. While we were able to validate several of the predicted chimeric RNAs with PCR and Sanger sequencing, some of the predicted SARS-Cov-2 specific chimeras may be due to the differences in genetic backgrounds from the largely divergent race demographics between the mostly European GTEx donors and the Chinese SARS-CoV-2 patients of this study.

Using binary expression profiles of chimeric transcripts and dimension reduction analysis, we were able to separate symptomatic from asymptomatic patients; however, further separation of disease groups was not as successful as similar approaches using standard gene expression profiles.^5^ In the future, the implementation of quantitative chimeric expression profiles with dimension reduction analysis may improve disease status cluster separation. Using the chimeric parental genes from every COVID-19 disease status group, we were able to generate unique gene set enrichments for each group. There was, however, an overlap of erythroid differentiation and motor behavior processes in the severe and critical groups as well as a general loss in pathway diversity represented in the enrichment results compared with the mild and asymptomatic groups (Fig. 3D). Interestingly, we have found that the most enriched processes of chimeric parental genes among symptomatic samples are associated with lipid or fatty acid oxidation while other recent studies have shown that oxidative stress is a major driver of COVID-19 pathogenesis.^41^^,^^42^ Consistent with the differential expression of the chimeric OS and TS transcript parental genes, reported here (Fig. 6E) as well as by Mukherjee et al, critical/severe COVID-19 patient-specific chimeras may highlight the genes under aberrant transcriptional or co-transcriptional regulation contributing to the dysregulation of erythrocyte dynamics and neuron dysfunction apparent in COVID-19 patients.^29^^,^^32^, ^33^, ^34^, ^35^

Furthermore, when comparing the 5′ and 3′ parental gene enrichment GO terms from GTEx whole blood with those from COVID-19 whole blood, we observed no overlap. GTEx whole blood 5′ parental gene GO terms mainly included leukocyte and neutrophil degranulation and activation pathways. Exocytosis was also found to be enriched in 5′ gene parental GO terms from GTEx whole blood, contrary to the endosomal and intracellular transport pathways found to be enriched in 5′ parental genes of COVID-19 whole blood chimeras. GTEx whole blood 3′ parental genes were also largely enriched in activation processes involved with immune response.

From the RNA binding protein motif analysis, we found an enrichment of sequences upstream of the chimeric splice acceptor site that contain a PTBP1 binding site. PTBP1's nucleocytoplasmic translocation is a well-documented response to viral infection that may be protective against coronavirus infection^26^ or necessary for viral replication.^27^^,^^28^^,^^36^ However, PTBP1 acts as a non-essential splice factor in the nucleus where it functions as both a splice repressor and splice activator.^37^^,^^38^ If PTBP1 translocates from the nuclear compartment to cytoplasm, there should be evidence for the loss of function for PTBP1's splicing role in the nucleus and consequently an effect on alternatively spliced mRNA isoforms found in the cell.^31^^,^^37^, ^38^, ^39^ Accordingly, PTBP1 knockdown in primary CD34 cells treated with erythropoietin (EPO) resulted in the detection of OS/TS chimeras, which were also shown to have a significant correlation with asymptomatic disease status in COVID-19 patients. While the literature supports a potential mechanism for PTBP1-dependent exclusion of the terminal SCO2 exon of OS/TS transcripts based on the proximal location of the PTBP1 binding sites,^31^ the detection of OS/TS chimeric transcripts in PTBP1 knockdown samples may be a consequence of increased transcription at that locus rather than divergent splice patterns. It is also possible that the detection of OS/TS transcripts is a consequence of changes in the OS/TS-positive versus OS/TS-negative cell populations. Further support for PTBP1's contribution to chimeric RNA generation and the COVID-19 patient phenotype comes from the induction of IFN signaling in CD34-derived erythroid cells upon PTBP1 knockdown, a phenomenon that appears to coincide with the presence of OS/TS chimeric transcripts in asymptomatic COVID-19 patients.^5^^,^^29^ The current analysis is based on a knockdown that results in a loss of function in both the nuclear and cytoplasmic compartments of the cell. Further interrogation is necessary to dissect the nuclear-specific loss of function phenotype that may be associated with coronavirus infections.

Using a reference made from human and SARS-CoV-2 genomes, we searched for virus-human chimeras that may indicate reverse transcription and integration into the human genome. We were unable to detect any evidence of these phenomena in human whole blood. Furthermore, we were only able to detect a single read that aligned to the SARS-CoV-2 genome from the whole blood RNA sequencing. The lack of detectable virus in the COVID-19 patient's whole blood suggests PTBP1's role in chimeric RNA generation may be downstream of cytokine signal transduction. Further interrogation is necessary to understand how PTBP1 cellular localization is influenced by cytokine signaling and its influences on the alternatively spliced transcriptome.

Our findings illustrate the significance of chimeric RNA interrogation in transcriptomic analysis studies. We discovered over 300 chimeric RNA transcripts in COVID-19 patient whole blood samples that were not present in GTEx whole blood samples, known to be free of SARS-CoV-2 infection. RNA binding motifs exhibited distinct enrichment patterns between chimeric RNAs from GTEx whole blood and those from COVID-19 whole blood. The findings suggest that PTBP1 interacting mRNA transcripts, such as chimeric OS and TS, may serve as valuable biomarkers for the early-stage presence of viral infection even when symptoms of infection are not observed and viral RNA is undetectable in the blood.

## Conflict of interests

The authors declared no competing interests.

## Funding

This work was supported by the 10.13039/100000002National Institutes of Health (USA) (No. R01GM132138 to H.L.).
