# Supplementary material for: Landscape of chimeric RNAs in COVID-19 patient blood
Source: Genes Dis. 2024 Jun 6;12(3):101348. doi: 10.1016/j.gendis.2024.101348 (PMC11919593; doi:10.1016/j.gendis.2024.101348)
Supplement: Multimedia component 1 [file mmc1.pdf]

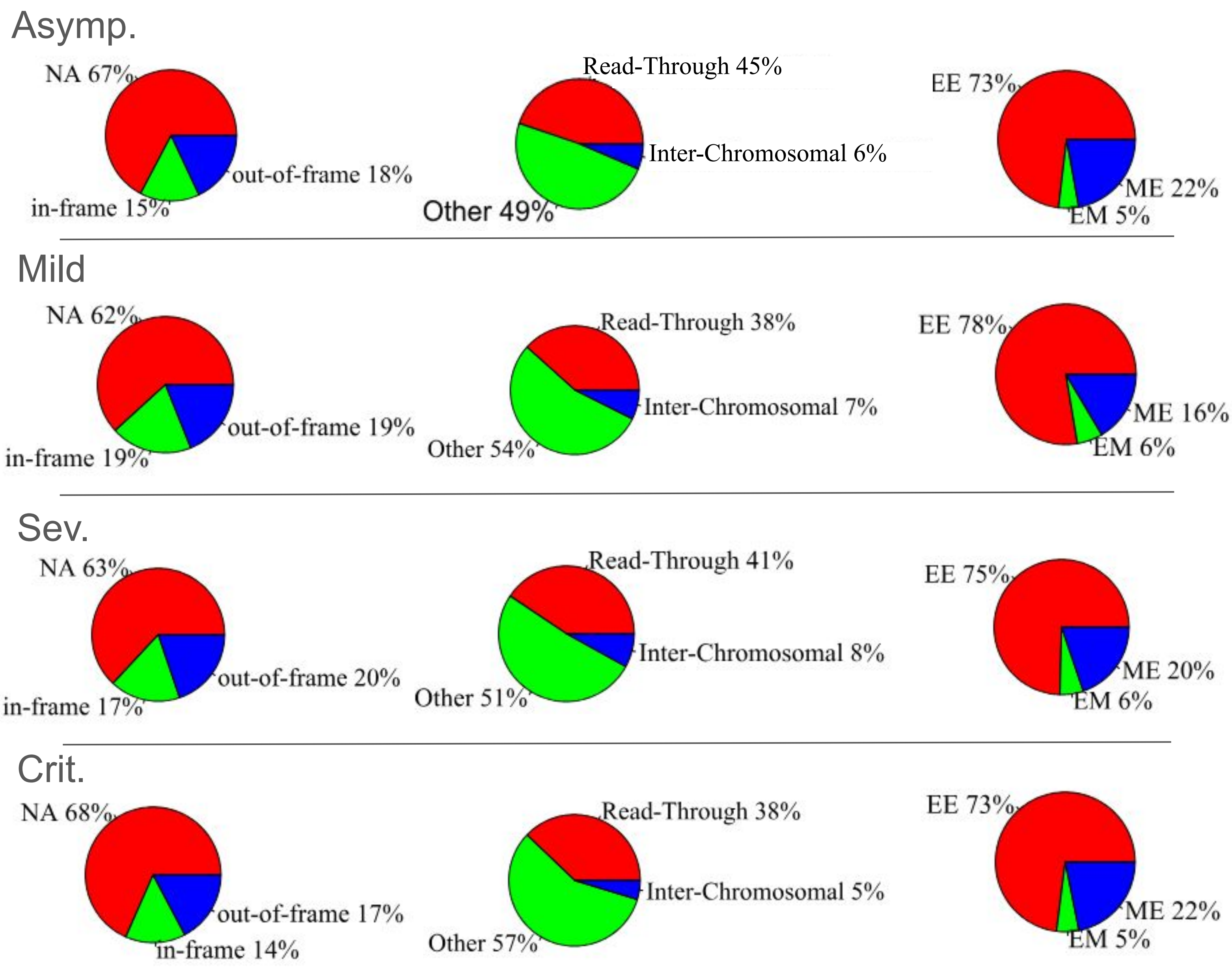

Supplemental Figure 1 - The distribution of chimeric RNAs found with 2 or more prediction software and after removal of M/M chimeras for each disease status group.

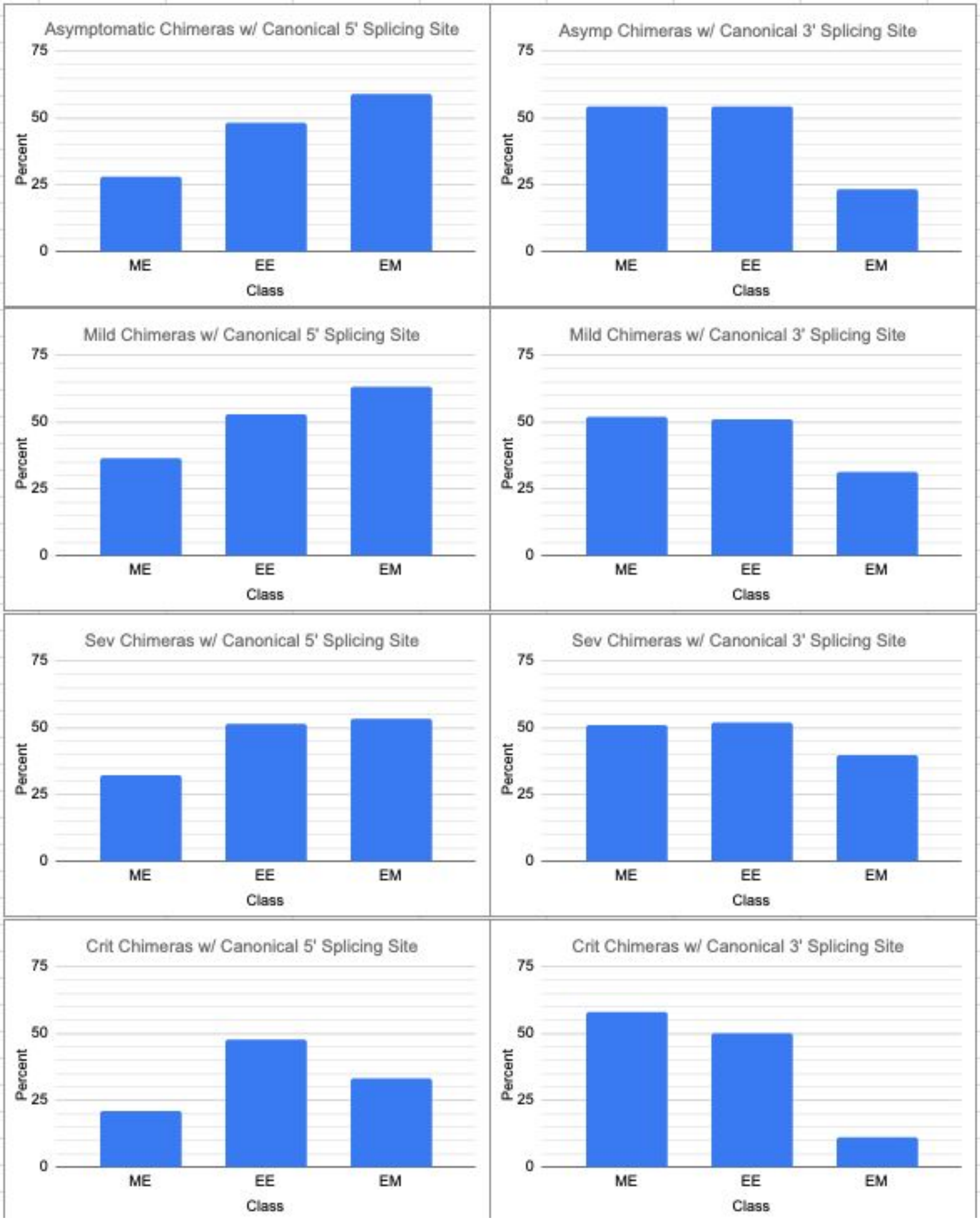

Supplemental Figure 2 - Percentage of chimeric RNAs harboring the canonical splicing donor sequence (AG/GT) at the 5′ junction for each disease status group (left). Percentage of chimeric RNAs harboring canonical splicing acceptor sequence (AG/G) at the 3′ junction for each disease status group (right).

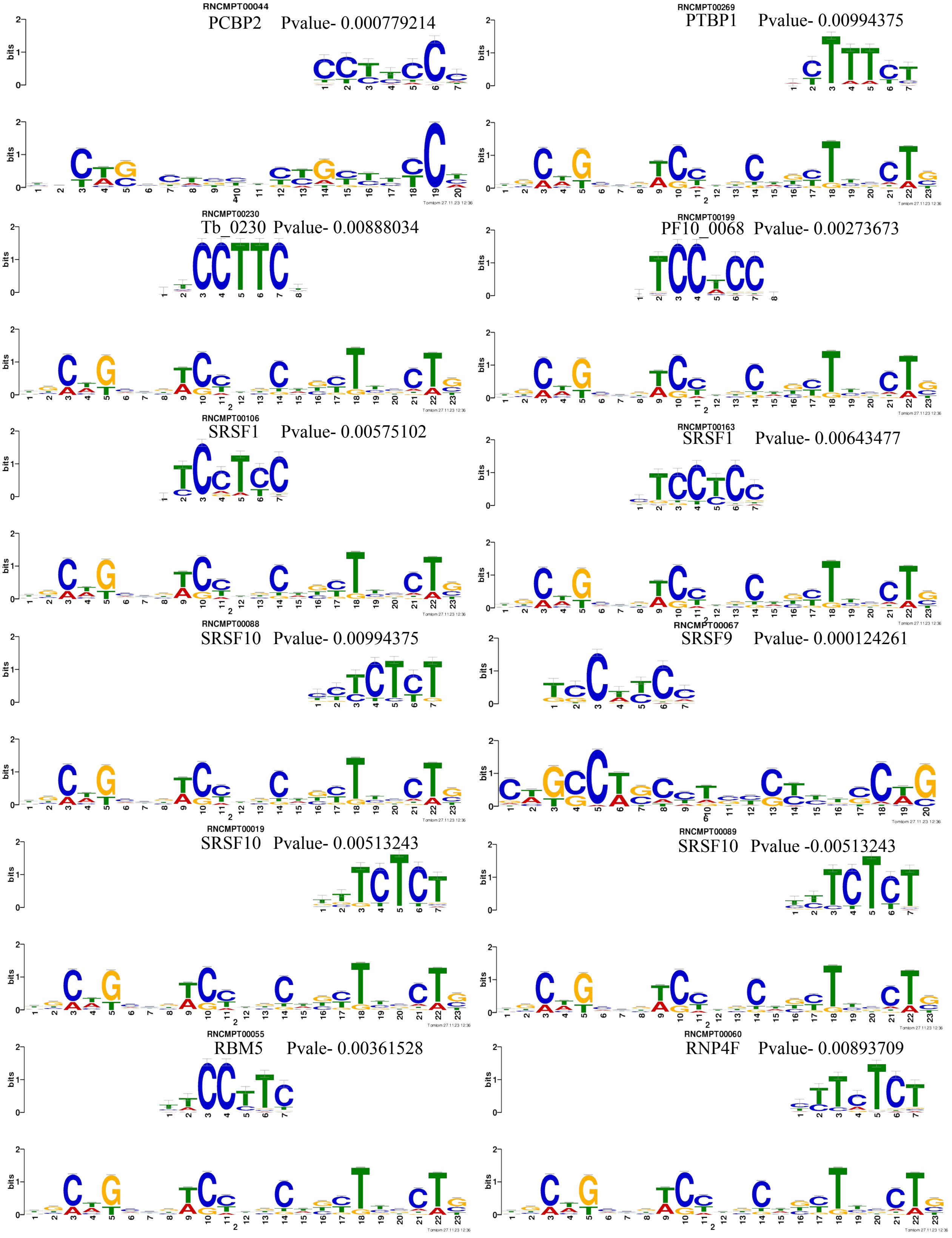

Supplemental Figure 3-A - RNA binding protein motif enrichment analysis with GLAM and TomTom of sequences upstream of 5' breakpoint. The sequence logos above show the enriched RNA binding protein motifs found above the top enriched reference sequences to which they aligned.

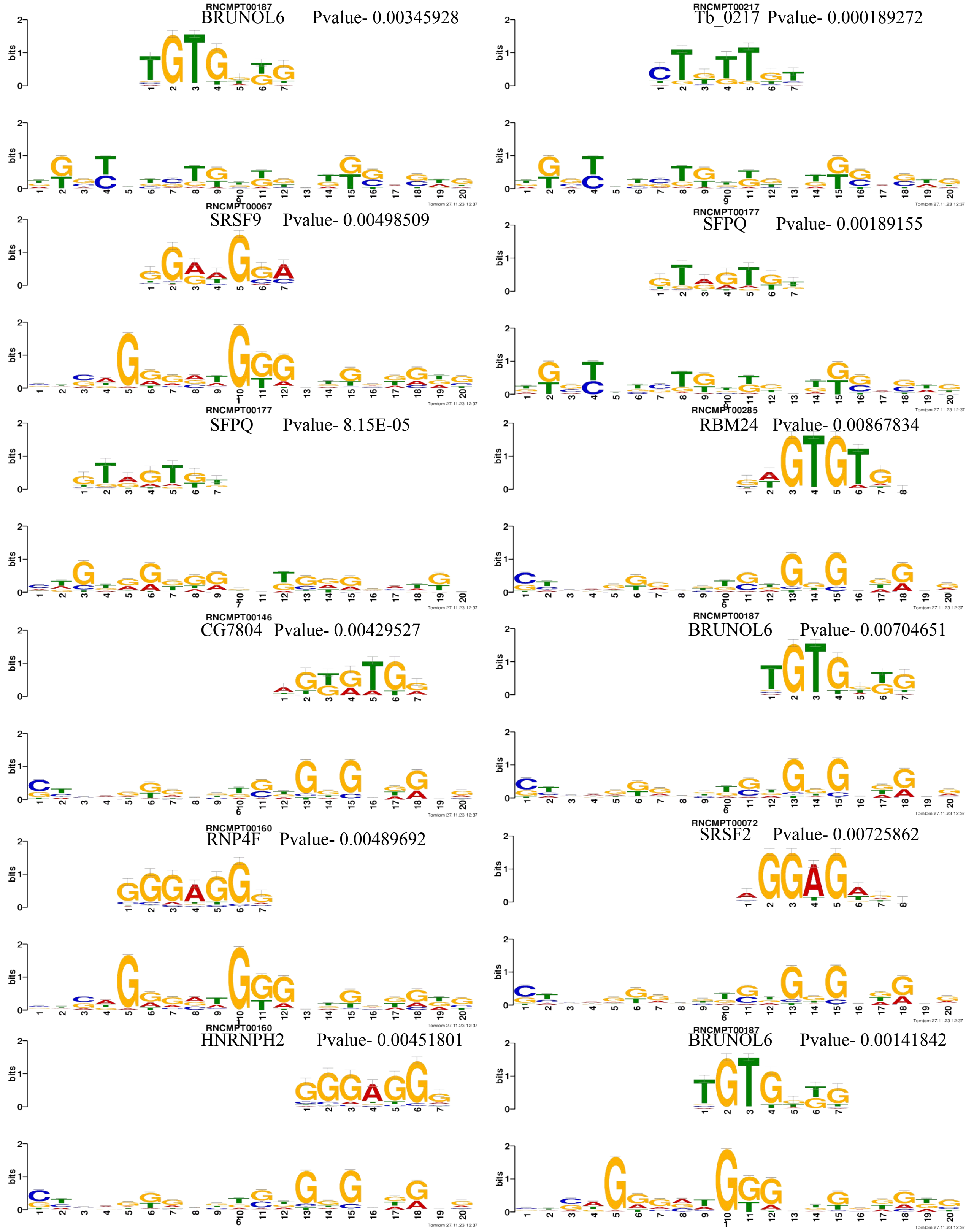

Supplemental Figure 3-B - RNA binding protein motif enrichment analysis with GLAM and TomTom of sequences downstream of 5’ breakpoint. The sequence logos above show the enriched RNA binding protein motifs found above the top enriched reference sequences to which they aligned.

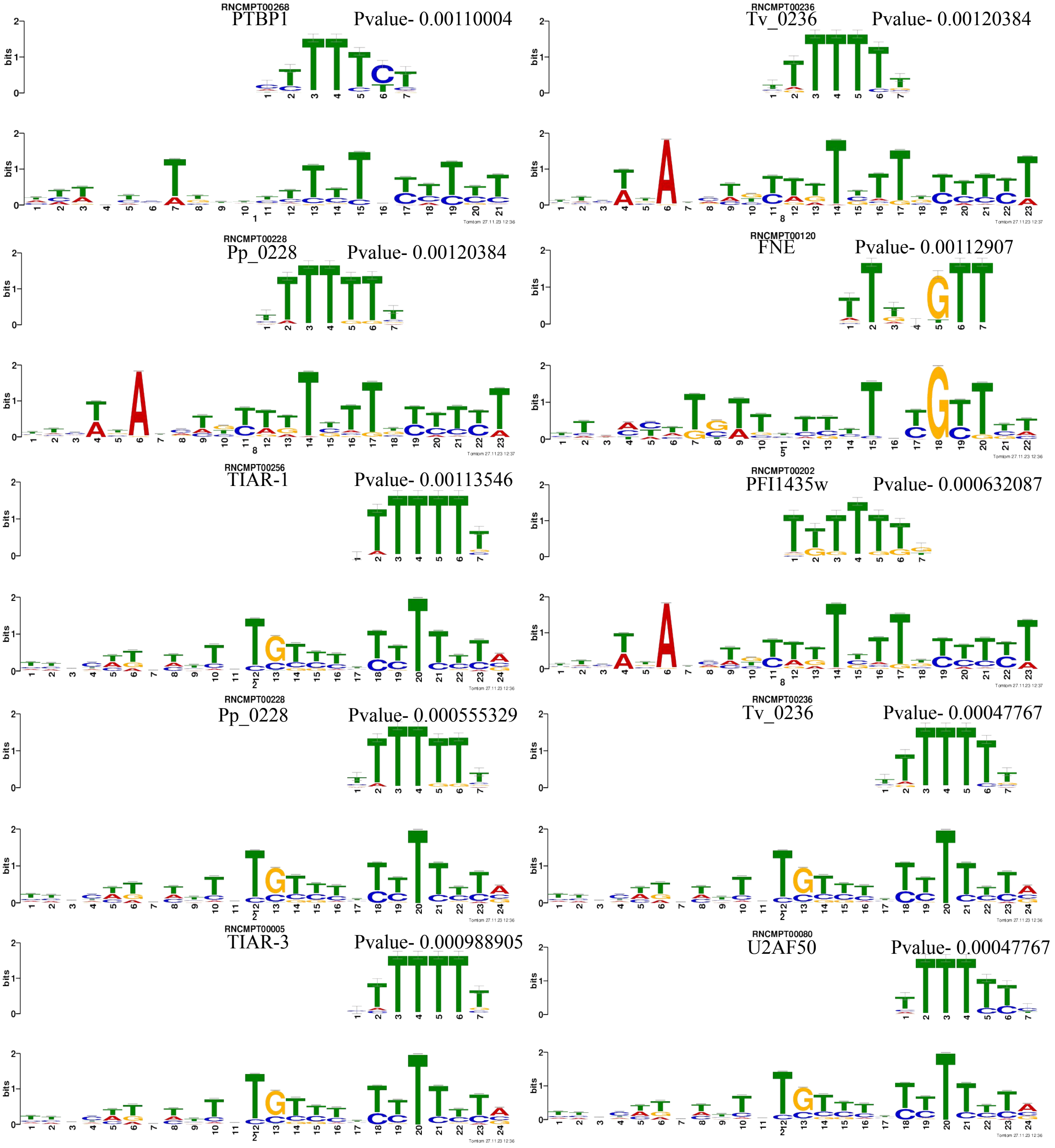

Supplemental Figure 3-C - RNA binding protein motif enrichment analysis with GLAM and TomTom of sequences upstream of 3' breakpoint. The sequence logos above show the enriched RNA binding protein motifs found above the top enriched reference sequences to which they aligned.

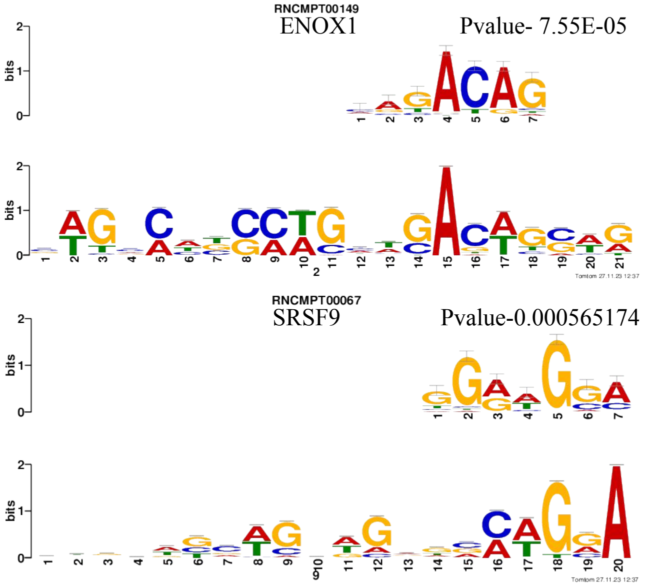

Supplemental Figure 3-D - RNA binding protein motif enrichment analysis with GLAM and TomTom of sequences downstream of 3' breakpoint. The sequence logos above show the enriched RNA binding protein motifs found above the top enriched reference sequences to which they aligned.
